# Protocol: a beginner’s guide to the analysis of RNA-directed DNA methylation in plants

**DOI:** 10.1186/1746-4811-10-18

**Published:** 2014-06-14

**Authors:** Huiming Zhang, Kai Tang, Bangshing Wang, Cheng-Guo Duan, Zhaobo Lang, Jian-Kang Zhu

**Affiliations:** 1 Department of Horticulture and Landscape Architecture, Purdue University, West Lafayette, IN 47907, USA; 2 Shanghai Center for Plant Stress Biology, Shanghai Institute of Biological Sciences, Chinese Academy of Sciences, Beijing, Shijingshan, China

**Keywords:** Epigenetics, RNA-directed DNA methylation, siRNA, Scaffold RNA, Pol IV, Pol V, Nuclei fractionation

## Abstract

**Background:**

DNA methylation is a conserved epigenetic mark that controls genome stability, development and environmental responses in many eukaryotes. DNA methylation can be guided by non-coding RNAs that include small interfering RNAs and scaffold RNAs. Although measurement of DNA methylation and regulatory non-coding RNAs is desirable for many biologists who are interested in exploring epigenetic regulation in their areas, conventional methods have limitations and are technically challenging. For instance, traditional siRNA detection through RNA hybridization requires relatively large amount of small RNAs and involves radioactive isotopes. An alternative approach is RT-qPCR that employs stem loop primers during reverse transcription; however, it requires a prerequisite that the exact sequences of siRNAs should be known.

**Results:**

By using the model organism Arabidopsis thaliana, we developed an easy-to-follow, integrative procedure for time-efficient, quantitative measurement of DNA methylation, small interfering RNAs, and scaffold RNAs. Starting with simplified nucleic acid manipulation, we examined DNA methylation levels by using Chop PCR (methylation-sensitive enzyme digestion followed by PCR), which allowed for fast screening for DNA methylation mutants without the need of transgenic reporters. We deployed a simple bioinformatics method for mining published small RNA databases, in order to obtain the nucleotide (nt) sequences of individual 24nt siRNAs within the regions of interest. The protocol of commercial TaqMan Small RNA Assay was subsequently optimized for reliable quantitative detection of individual siRNAs. We used nested qPCR to quantify scaffold RNAs that are of low abundance and without Poly-A tails. In addition, nuclei fraction enables separation of chromatin-associated scaffold RNAs from their cognate non-scaffold transcripts that have been released from chromatin.

**Conclusions:**

We have developed a procedure for quantitative investigations on nucleic acids that are core components of RNA-directed DNA methylation. Our results not only demonstrated the efficacy of this procedure, but also provide lists of methylation-sensitive restriction enzymes, novel DNA methylation marker loci, and related siRNA sequences, all of which can be valuable for future epigenetic studies. Importantly, step-by-step protocols are provided in details such that the approaches can be easily followed by biologists with little experience in epigenetics.

## Background

**Figure 1:**
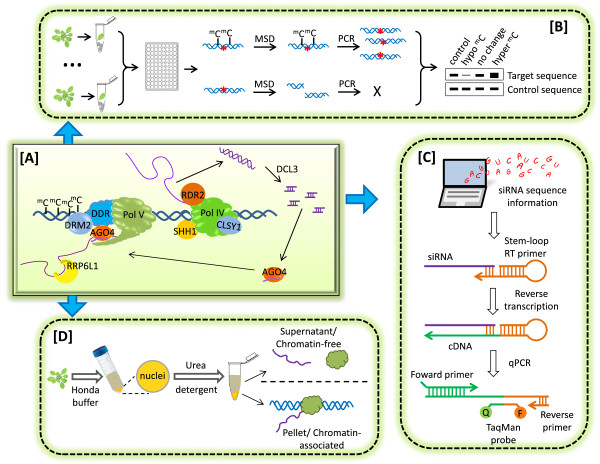
**An integrative procedure for studying nucleic acids in RdDM. [A]** A model of RdDM in Arabidopsis. Briefly, Pol IV initiates siRNA production by producing single-stranded non-coding RNAs, while Pol V synthesizes scaffold RNAs that recruits Argonaute-associated siRNAs. The complimentary pairing between the two types of non-coding RNAs, as well as physical interactions between proteins in the methylation complex, results in targeting of the methyltransferase DRM2 to RdDM loci. For simplicity, not all known RdDM components are shown. **[B]** Chop PCR-based screen for mutants showing abnormal DNA methylation patterns. Red asterisks represent enzyme restriction sites. Cytosine methylation overlapping restriction sites will protect DNA from methylation-sensitive digestion (MSD), while enzymatic cleavage results in failure of subsequent PCR amplification of the target sequences. Note that the enzyme McrBc is special in that it cleaves methylated- but not unmethylated DNA sequences (see also Additional file 2: Table S1). **[C]** RT-qPCR detection of siRNAs by using stem-loop primers during reverse transcription. **[D]** Schematic procedure of nuclei fractionation, which separates chromatin-associated RNAs from RNAs that are not associated with chromatin. See Methods for details.

DNA methylation is a major epigenetic mark that can confer transcriptional silencing of genes and transposable elements. RNA-directed DNA methylation (RdDM), which involves complimentary pairing between small RNAs [including small interfering RNAs (siRNAs) and piwi-associated RNAs (piRNAs)] and scaffold RNAs, has been established in plants and mammals [1-5]. DNA methylation is subject to dynamic regulation during establishment, maintenance, and removal in response to developmental and environmental cues [6]. In order to understand epigenetic regulation, it is important to characterize the histone modification patterns and to determine the levels of DNA methylation and small RNAs, as well as of scaffold RNAs that are chromatin-associated long non-coding RNAs (Figure 1A). This study focuses on assays for DNA methylation, small RNAs and scaffold RNAs, since methods for characterizing histone modification patterns such as chromatin immunoprecipitation (ChIP) have been covered extensively [7,8]. Genome-wide DNA methylation and siRNA accumulation patterns can be precisely profiled by next-generation-sequencing. At individual loci, conventional methods for obtaining data on DNA methylation and non-coding RNAs include bisulfite sequencing for DNA methylation, RNA hybridization for siRNAs, and RT-PCR (reverse transcription followed by polymerase chain reaction) for scaffold RNAs [9,10]. Although commonly used, these methods have limitations. Bisulfite sequencing is costly and time-consuming, and the assay requires considerable experience in several important steps of the experimental procedure including designing suitable primers. Northern blotting of siRNA requires large amounts of small RNA input and involves radioactive isotopes. RT-PCR is semi-quantitative and, if applied alone, does not distinguish scaffold RNAs from those transcripts that have been released from chromatin. To overcome these limitations, we used the model organism *Arabidopsis thaliana* to develop PCR- or qPCR-based methods for studying nucleic acids in RdDM. These optimized approaches form an integrative procedure that can be followed by biologists with little experience in epigenetic studies (Figure 1).

## Results and discussion

The first part of this procedure is to detect alteration in DNA methylation levels. Alternative to bisulfite sequencing, DNA methylation levels in different samples can be compared by Chop PCR, in which methylation-sensitive enzyme digestion of DNA is followed by PCR amplification of the target sequence (Figure 1B). By using Chop PCR, we developed a method for epigenetic mutant screen that does not need a transgenic reporter gene. A large population of Arabidopsis mutants is commercially available from seed stock sources such as ABRC (Arabidopsis Biological Resource Center) and can be used for Chop PCR-based mutant screen. Genomic DNA samples were prepared by a simple home-made recipe that is suitable for simultaneous DNA extraction from multiple samples. We used the methylation-sensitive restriction enzyme Hae III and examined DNA methylation at *AtSN1*, a known RdDM target locus. Hundreds of Arabidopsis mutants were screened, resulting in discovery of several DNA hypomethylation mutants including *atrrp6l1* alleles that were recently characterized [11]. In addition to Hae III, we have examined some other methylation-sensitive restriction enzymes and correspondingly demonstrated the efficacy of these enzymes in examining several RdDM marker loci in Arabidopsis (Additional file 1: Figure S1; Additional file 2: Table S1). Different restriction enzymes can be used to assay methyaltion in the different sequence contexts, i.e., CG, CHG, and CHH (H represents A, T, or C). Although Chop PCR examines less cytosine than bisulfite sequencing, observation of DNA hypomethylation by Chop PCR in multiple marker loci is sufficient to demonstrate an epigenetic function. Quantification of the difference in DNA methylation levels can be achieved by Chop qPCR (Additional file 1: Figure S1B). Parallel amplification of the marker locus from non-digested DNA samples is commonly used to show loading controls. However, we recommend amplifying DNA sequences without restriction sites, from the same digested DNA samples, as internal controls, because experimental accuracy can be increased while the amount of required DNA can be reduced. Compared to traditional forward genetic screens that involve transgenic reporter genes, Chop PCR-based screen does not require generation of a transgenic reporter line and the subsequent mutated population, neither required is the process of map-based cloning that can be time-consuming.

The second part of this procedure is quantification of siRNAs (Figure 1C). The levels of siRNAs can be semi-quantitatively detected by RNA hybridization, which typically uses ≥20 μg RNAs that are enriched in sizes smaller than 200 bp [12]. The whole process takes several days from RNA extraction to final results. Alternative to RNA hybridization, small RNAs can be detected by RT-qPCR that employs stem loop primers during reverse transcription [13]. This approach takes only a few hours starting from RNA extraction and involves the patented TaqMan Small RNA Assays. However, the protocol of TaqMan Small RNA Assays, which was initially implemented to detect miRNAs, does not readily enable detection of lower abundance small RNAs such as 24 nt siRNAs that mediate RdDM. In addition, these PCR-based quantitative measurements require, as a prerequisite, knowing the exact sequences of small RNAs; meanwhile mining published small RNA datasets for such information typically requires skills in bioinformatics. Therefore we developed a simple bioinformatics method for selecting siRNAs to quantify and optimized the TaqMan Small RNA Assays protocol for detecting 24 nt siRNAs.

To obtain nucleotide sequences for 24 nt siRNAs from the regions of interest, we searched small RNA sequencing databases. This can be done by BLAST searches of published small RNA databases, such as Zhang et al. [14], for siRNAs that match the regions of interest. However, such analysis requires skills in bioinformatics. We thus provide an alternative analysis approach that can be applied in any laboratory, by using Arabidopsis *AtSN1* locus as an example. *AtSN1* locates within 1 kb downstream of the gene At3g44006 [9]. Browsing *At3g44006* and its downstream region in IGB (Integrated Genome Brower) identified *AtSN1* as *At3TE63860*, which is a transposable element that starts at 15794808 and ends at 15794619. Arabidopsis small RNA nucleotide sequence information with genomic coordinates is available in the UDelaware Small RNA database [15]. Since 24 nt siRNAs are proposed to pair with scaffold RNAs [1-4], we screened *AtSN1* small RNAs for 24 nt siRNAs that are annotated as Waston strand, i.e., template DNA strand. This means that these siRNAs have the same sequences as the template DNA strand (despite the U/T difference) and thus can potentially pair with scaffold RNAs. To increase the chance of detecting individual siRNAs, these 24 nt siRNAs were subjected to a second screen for those that showed abundance of ≥ 1 hit per million reads, according to the UDelaware Small RNA database, in the wild type *Col-0* ecotype. Among these candidates, one siRNA exhibited sequence homology to a 43 nt probe that had been used for detecting *AtSN1* siRNAs by Northern blots [10]. Thus we chose this siRNA for customer TaqMan Small RNA Assay design.

**Figure 2:**
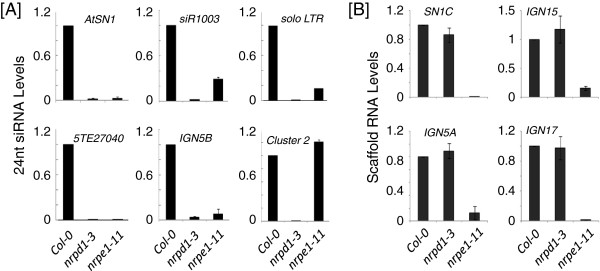
**Quantitative measurement of 24nt siRNAs and scaffold RNAs in Arabidopsis. [A]** Quantification of individual 24nt siRNAs by TaqMan small RNA assays. The levels of siRNAs were normalized by using *snoR101* as internal control. **[B]** Quantification of Pol V-dependent scaffold RNAs by gene/scaffold RNA-specific RT-qPCR. *Actin2* was used as internal control. Error bars indicate SD, n = 3 technical replicates. All experiments were performed with at least two biological repeats. Representative images are shown.

The manufacture-recommended protocol of TaqMan Small RNA Assay suggests using 1-10 ng total RNA per reaction. We were able to observe stress-induced regulation of miRNAs by using ≥ 50 ng total RNA as input (Additional file 1: Figure S2). However, most 24 nt siRNAs that we examined were undetectable under similar condition. We found that 400 ng small RNA (the ≤200 bp fraction of total RNA), instead of total RNA, per RT reaction is sufficient for consistently detecting all 24 siRNAs that we tested, including the *AtSN1* siRNA (Figure 2A). We used *snoR101*, a small nucleolar RNA, as the internal control to ensure equal RNA input. During the RT reactions, we mixed RNA with 2 μL 20X (manufacture-defined concentration) siRNA RT primer together with 2 μL *snoR101* RT primer, instead of using 3 μL siRNA RT primer alone as suggested by the manual. Subsequently, the levels of siRNA and *snoR101* were independently measured by quantitative PCR. Because siRNA and *snoR101* are simultaneously reverse transcribed in the same tube, the level of *snoR101* reflects RT efficiency of each sample, allowing for elimination of false results that may be caused by different RT efficiency between samples.

The effectiveness of using TaqMan Assays to quantify 24nt siRNA levels was confirmed by using the Arabidopsis mutants *nrpd1-3* that is defective in RNA polymerase Pol IV, which is required for biogenesis of almost all 24nt siRNAs [14], and *nrpe1-11* that is defective in Pol V, of which mutation decreases the levels of some Pol IV-dependent siRNAs [16]. Compared to wild type plants (*Col-0*), *nrpd1-3* exhibited depletion of all examined siRNAs including *AtSN1*, *siR1003*, *solo LTR*, *5TE27040*, *IGN5B*, and *Cluster 2* (Figure 2A). The *nrpe1-11* mutant also showed substantial reduction in the levels of the examined siRNAs except *Cluster 2* (Figure 2A), which is known to be Pol IV-dependent and Pol V-independent [17]. The patterns observed in our RT-qPCR measurements are consistent with published RNA hybridization results [10,17] and whole genome small RNA sequencing results [18]. Sequences of these individual 24nt siRNAs are listed in Additional file 2: Table S2. Designing siRNA probes requires only submitting individual siRNA sequences to the manufacturer website of the patented TaqMan Assays.

The third part of studying RdDM nucleic acids is to quantitatively measure chromatin-associated scaffold RNAs. In Arabidopsis, Pol V transcription generates scaffold RNAs that are of low abundance and are devoid of poly-A tail [9]. Therefore we used scaffold RNA-specific primers during reverse transcription for the first-strand cDNA synthesis, and subsequently performed nested qPCR. Such an approach successfully measures Pol V-transcribed scaffold RNAs, as demonstrated by comparison between *Col-0* and *nrpe1-11* (Figure 2B). Although the levels of scaffold RNAs are in proportion to the pool of total Pol V transcripts, sometimes nuclei fractionation is needed to distinguish chromatin-associated scaffold RNAs from those Pol V transcripts that have been released from chromatin (Figure 1D), as was the case in our recent study of AtRRP6L1 that functions in retention of Pol V transcripts at chromatin [11]. Nuclei can be isolated with Honda buffer and subsequently lysed in the presence of urea and detergent, followed by centrifugation that sediments chromatin-associated RNAs in the pellet (chromatin-associated fraction), while leaving released RNAs in the supernatant (chromatin-free fraction) (Figure 1D). As an indication of successful nuclei fractionation, genomic DNA should be nearly absent in the chromatin-free fractions (Additional file 1: Figure S3).

To provide an easy-to-follow procedure, experimental details are described step by step in the Methods.

## Conclusions

To summarize, we have developed an integrative procedure for studying nucleic acids that are central players in RNA-directed DNA methylation and other epigenetic regulation (Figure 1). The results not only demonstrated the efficacy of this procedure (Figure 2; Additional file 1: Figure S1-S3), but also provide a list of novel RdDM marker loci (Additional file 2: Table S1) and the related methylation-sensitive restriction enzymes (Additional file 2: Table S1), as well as the nucleotide sequences of a list of individual 24nt siRNA (Additional file 2: Table S2), which can be valuable for future epigenetic studies. Moreover, this procedure requires little previous experimental experience in epigenetics or bioinformatics, and thus can broadly benefit the scientific community.

## Methods

### I. Chop-PCR/qPCR

#### <I-1> Preparation of genomic DNA samples

1. Prepare 1.5 mL eppendorf tubes, each containing 3 Chrome steel beads (BioSpec Products, Inc, Cat.# 11079132C).

* Note: Steps 1 to 3 are optimized for preparing multiple samples. Start from Step 4 if samples are homogenized by other methods.

2. Take one or two mature leaves and put into labeled tubes. Make sure the tubes are tightly capped.

3. Freeze tubes in liquid nitrogen shortly, then shake tubes vigorously in a box to shatter the samples.

*Note: Be aware that a capped tube with liquid nitrogen in it will pop open or even explode under room temperature.

4. Add 500 μL 2% CTAB (Cetyltrimethyl ammonium bromide) solution to each tube (or per ~100 mg sample if homogenized by other methods), then incubate at 65°C for 30-60 min.

* Note: To make 100 mL CTAB solution (modified from [19]): 2g CTAB, 10 mL 1 M Tris-HCl (pH = 7.5), 28 mL 5 M NaCl, 4 mL 0.5 M EDTA (pH = 8.0), H_2_O.

5. Add 500 μL chloroform, then votex vigorously and centrifuge at ≥ 12000 g at room temperature for 10 min.

6. Transfer aqueous phase into new tubes, then mix with equal volume isopropanol.

7. Store at -20°C for ≥ 30 min.

8. Centrifuge at ≥ 12000 g at room temperature for 10 min.

9. Wash the pellet with 0.5 mL 70% ethanol.

10. Air dry pellets and dissolve in 50 μL nuclease-free water (we use autoclaved double-distilled water).

11. Proceed to steps in < I-2 > for Chop-PCR if doing mutant screening. Alternatively, for Chop-qPCR, continue with DNA purification as described below.

* Note: Although DNA samples extracted by this way have RNA contamination, they can be used for rough estimations of DNA methylation levels during initial mutant screen. RNase treatments should be performed for Chop qPCR and for semi-quantitative Chop PCR that is used for the purposes other than rough estimation.

12. Add 2 μL RNase A (10mg/mL) per 500 μL DNA solution, mix well and incubate at 37°C for 1 hr.

* Note 1: Concentration of DNA solutions can vary depending on the yield of crude extraction using CTAB solution.

* Note 2: If making RNase A solution from powders, boiling the solution for 15–20 minutes usually eliminate most of its DNases.

13. Add equal volume *phenol: chloroform: isoamyl alcohol* (24:1:1, pH 8.0), vortex vigorously for 5 sec, then centrifuge at ≥ 12000 g at room temperature for 10 min.

14. (Optional) Transfer the aqueous phase to a new tube, repeat Step 2.

15. Transfer the aqueous phase to a new tube, add equal volume *phenol: chloroform*, vortex vigorously for 5 sec, then centrifuge at ≥ 12000 g at room temperature for 8 min.

16. Transfer the aqueous phase to a new tube, add 2 volume ethanol, 0.1 volume NaOAc (pH 5.2, 3 M), mix well.

17. Store at -20°C for ≥ 30 min.

18. Centrifuge at ≥ 12000g at room temperature for 10 min.

19. Wash the pellet 2X with 0.5 mL 70% ethanol.

20. Air dry pellets and dissolve in nuclease-free water.

#### <I-2 *>* Enzymatic DNA digestion and PCR/qPCR

1. Measure genomic DNA concentration using Nanodrop.

2. Digest equal amount (typically 0.5 to 1 μg) of genomic DNA with a methylation-sensitive enzyme in a 20 μl reaction mixture.

* Note: While following manufacture protocols to set up reactions for each enzyme, the digestion time can be extended to overnight.

3. After digestion, check DNA methylation levels at the enzyme-targeted marker locus by qPCR or PCR. For semi-quantitative PCR,use 1 μL of the digested DNA as template in a 20 μl reaction mixture that contains 0.15 unit of commercial Taq DNA polymerase. Run a DNA gel (1.2% agarose) to compare the PCR results from different samples. For qPCR, prepare 20 μl reaction mixture following manufacture protocols and use 1 μl of the digested DNA as template per reaction.

* Note 1: DNA input amounts in different samples should be normalized by amplifying an internal control sequence that does not contain the restriction site of the enzyme used. Alternatively, non-digested DNA can be used as PCR/qPCR templates for input normalization.

* Note 2: In semi-quantitative PCR, too many PCR cycles will probably yield in saturated amplification and thereby mask small differences in template inputs. For optimized observation of differences, PCR can be repeated with increased or decreased cycle numbers after initial assessment. A positive control, e.g., the Pol IV mutant *nrpd1-3*, should be examined in parallel to the wild type.

Optional: For Chop-qPCR, follow steps 4 to 10 to analyze the results

4. Export qPCR results to Excel file.

5. Calculate average Ct value for the non-digested control in each sample.

6. Calculate “ΔCt = Ct of the examined amplicons – average Ct of *the non-digested control*”.

7. Calculate average ΔCt value of one sample (e.g., wild type plants).

8. Calculate the comparative Ct values (ΔΔCt) for other samples (e.g., mutant plants). ΔΔCt = ΔCt _(mutant)_ – average ΔCt _(wild type)_.

9. Calculate relative abundance of the amplicons by using this function: “= 2^(-ΔΔCt)”.

10. Calculate average of relative abundance of the amplicons; and calculate standard deviation by using the function “=STDEV(values to be calculated)”.

### II. siRNA RT-qPCR

#### <II-1> Isolation of small RNAs

1. Homogenize ≤100 mg plant tissues in liquid nitrogen by using mortar and pestle. Transfer the powder into a 1.5 mL eppendorf tube (pre-chilled in liquid nitrogen) and add 1 mL RNAzol® RT (Molecular Research Center Inc)

2. Add 0.4 mL water per 1 mL of RNAzol® RT. Mix well and store at room temperature for 5-15 min.

* Note: Autoclaved distilled water is good enough for this step.

3. Centrifuge the mixture ≥12,000 g at room temperature for 15 min.

* Note: this step precipitates DNA, protein, and polysaccharide.

4. Transfer the supernatant to a new tube and add 0.4 mL 75% ethanol per 1 mL of supernatant. Mix well and store at room temperature for 10 min.

5. Centrifuge ≥12,000 g for 8 min at room temperature or at 4°C.

* Note: this step pellets RNA > 200 bp.

6. Transfer the supernatant to a new tube and add 0.8 volume of isopropanol. Mix well and store at 4°C for 30 min.

* Note: the mixture can be stored at -20°C for longer time.

7. Centrifuge ≥12,000g for 15 min at room temperature or at 4°C.

8. Wash the pellet with 70% isopropanol twice.

* Note: After removing the bulk isopropanol solution, centrifuge briefly and use pipette to remove the residual liquid. By doing this, RNA pellets dissolved without drying perform well in the downstream RT-PCR reactions.

9. Dissolve the pellet in 20-40 μL RNase-free water, without drying, in RNase-free water. Keep on ice and proceed to reverse transcription, or store at -80°C.

#### <II-2> Quantitative measurement of siRNAs by RT-qPCR

1. Search small RNA databases to determine sequences of siRNAs to be examined (see main text for an example).

* Note: Additional file 2: Table S2 lists some Arabidopsis 24nt siRNAs that have been detected by RT-qPCR in this study.

2. Submit siRNA sequences to Life Technologies for TaqMan Small RNA Assay design by following the online instructions.

3. Place the order after the designing has been completed.

* Note: we suggest downloading the TaqMan Small RNA Assay protocol at this point, as well as to order the TaqMan MicroRNA Reverse Transcription Kit and the TaqMan Universal PCR Master Mix as recommended in the protocol.

4. Use TaqMan MicroRNA Reverse Transcription Kit to prepare reverse transcription (RT) master mixture and keep on ice. The following recipe is for one reaction:

100 mM dNTPs 0.15 μL

MultiScribe TM Reverse Transcriptase (50 unit/μL) 1.0 μL

10X Reverse Transcription Buffer 1.5 μL

RNaseOut (40 unit/μL) 0.9 μL

RNase-free Water 4.26 μL

Total Volume: 7 μL

5. In an RNase-free PCR tube, add 400 ng small RNA in a volume of 5 μL. Then add 2 μL *snoR101* RT primer and 2 μL siRNA RT primer. Mix well gently.

400ng small RNA is sufficient for consistently detecting all 24 nt siRNAs that we have examined. For certain abundant siRNAs such as siR1003 that originates from 5S rDNA repeats, 100 ng small RNA is sufficient.

6. Incubate the RNA-primers mixture at 85°C for 5 min, followed by 60°C for 5 min. Then put the mixture on ice immediately and for at least 1 min.

7. Add 7 μL RT mixture to each RNA-primers mixture. Mix well gently. Keep in ice for 5 min.

8. Perform RT reaction using the following conditions consecutively: 16°C for 30 min, 42°C for 30 min, 85°C for 5 min. Keep the samples on ice after RT reaction.* Note: Because TaqMan stem-loop primer recognizes a specific short sequence and the qPCR reverse primer is complementary to the stem-loop RT primers (see Figure 1C in main text), genomic DNA contamination, if any, will not be a problem for this experiment. Thus no-RT control is unnecessary. In fact, we did not detect qPCR signals even using genomic DNA.

9. Prepare the qPCR mixture as the following:

TaqMan Small RNA Assay (20×) 1.0 μL

cDNA from RT reaction (Step 8) 1.2 μL

TaqMan Universal PCR Master Mix II (2×) 10.0 μL

Nuclease-free water 7.8 μL

Total Volume: 20 μL

* Note 1: run qPCR individually for *snoR101* and the siRNA and perform 3 technical repeats.

* Note 2: The patented TaqMan Small RNA Assays provide qPCR primers in the tubes labeled as “TaqMan Small RNA Assay (20×)” without giving primers information.

10. Perform qPCR in a real-time PCR instrument using the following program:

Step I: 50°C (optional) 2 min

Step II: 95°C (Enzyme activation) 10 min

Step III: 95°C (Template denaturing) 15 sec

Step IV: 60°C (Anneal/extend) 1 min

Step V: go to Step III, 39 times (totally 40 cycles)

Step VI: End

* Note: TaqMan Small RNA Assay PCR signals will be detected in the FAM channel by the qPCR instrument.

11. Export results to Excel file.

12. Calculate average Ct value for snoR101 in different replicates.

13. Calculate “ΔCt = Ct of siRNA – average Ct of *snoR101*” from the same RT reaction.

14. Calculate average ΔCt value of one RNA sample (e.g., wild type plants).

15. Calculate the comparative Ct values (ΔΔCt) for other samples (e.g., mutant plants). ΔΔCt = ΔCt _(mutant)_ – average ΔCt _(wild type)_.

16. Calculate relative siRNA abundance by using this function: “= 2^(-ΔΔCt)”.

17. Calculate average of relative siRNA abundance; and calculate standard deviation by using the function “=STDEV(values to be calculated)”.

* Note: Mean value and standard deviation can also be calculated based on multiple biological replicates. The expression patterns, e.g., release of transcriptional silencing, should always be the same for different biological replicates, although the standard deviation between different biological replicates is usually greater than that of technical repeats from one single biological replicate.

### III. Scaffold RNA RT-qPCR

#### <III-1> Quantitative measurement of scaffold RNAs without nuclei fractionation

1. Extract total RNA by using Trizol (Invitrogen) following the manufacturer’s protocol.

* Note: Scaffold RNAs may be a few hundred nucleotides, so for this experiment we do not recommend using those commercial RNA extraction kits that preferentially extract RNA > 200 bp.

2. Remove DNA contamination in the RNA samples by using TURBO DNA-free™ (Ambion).

* Note: We typically treat 10 μg RNA with TURBO DNase in a 20 μL reaction, and use maximum amount of RNA during RT reaction while following the manufacturer RT protocol.

3. Perform RT reaction by using SuperScript™ III First-Strand Synthesis SuperMix (Invitrogen). In each reaction, use mixed gene/scaffold RNA-specific primers (the scaffold RNA and the internal control *ACTIN2*). For a 10 μL reaction, use 0.5 μL pre-mixed primers (2 mM each).

* Note 1: We have tried using some other RT kits and found that, in our hands, SuperScript works best for low-abundance non-coding RNA detection.

* Note 2: We recommend using the same amount of RNA for each RT reaction. We typically use 1 μg total RNA for a reaction in 10 μL.

* Note 3: No-RT control reactions can be performed by making RT mixtures as describe above but without reverse transcriptase. This will ensure no false qPCR signals from DNA contamination in RNA samples.

4. Perform qPCR by using SYBR® Green Master Mix. PCR for scaffold RNA and *ACTIN2* should be separated.

Step I: 95°C 3 min

Step II: 95°C 10 sec

Step III: Tm 30 sec

Step IV: go to Step II 39 times + plate read (totally 40 cycles)

Step V: increase from 65°C to 95°C, increment 0.5°C for 5 sec, plus plate read

Step VI: End

* Note 1: Because cDNAs are synthesized by using gene-/scaffold RNA-specific primers, PCR amplification that follows has to be nested PCR, i.e., positions of the qPCR primers on the DNA coding strand should be upstream of the gene-/scaffold RNA-specific RT primers.

* Note 2: A single peak in the melt curve generated by Step V should indicate the specificity of the primers. This can also be examined by DNA gel electrophoresis.

5. Use *ACTIN2* as internal control to analyze the results (Refer to Steps *V-11 to V-17* for details).

* Note: By starting with equal amount of RNA, different samples should show similar Ct value for *ACTIN2* in qPCR reactions. Alternatively, another housekeeping gene can be examined to ensure similar RNA quality.

#### <III-2> Quantitative measurement of scaffold RNAs with nuclei fractionation

1. Prepare Honda buffer (0.4 M Sucrose, 2.5% Ficoll, 5% Dextran T40, 25 mM Tris-HCl, pH 7.4, 10 mM MgCl2, 0.5% Triton X-100, 0.5 mM PMSF, 0.1% pepstatin A, 0.1% aprotinin, 10 mM β-mercaptoethanol, 8 unit/mL RNaseOUT) (modified from [9]).

* Note: add PMSF, pepstatin A, aprotinin, β-mercaptoethanol, and RNaseOUT before use.

2. Grind 3 gram ~12-day-old plants with liquid nitrogen.

* Note: We recommend using the same amount of plant materials for each sample.

3. Transfer the power to a 50 mL Falcon tube prefilled with 15 mL cold Honda buffer. Homogenize by vortex vigorously. Keep the tubes in ice for 15-30 min.

4. Filter the homogenate through 4 layers of Miracloth, followed by centrifugation at 1500 g at 4°C for 5 min.

5. Re-suspend the pellet in 1 mL Honda buffer, then transfer to a new 1.5 mL eppendorf tube, followed by centrifugation at 1800 g at 4°C for 5 min to pellet the nuclei.

* Note: Save the supernatant for cytosol analysis if wished.

6. Gently rinse the nuclei pellet with ice-cold wash buffer (1X PBS, 1 mM EDTA, 8 unit/mL RNaseOUT).

7. Re-suspend the pellet in 0.5 mL prechilled glycerol buffer (20 mM Tris-HCl, pH 7.9, 75 mM NaCl, 0.5 mM EDTA, 0.85 mM DTT, 50% glycerol, 0.125 mM PMSF, 0.1% pepstatin A, 0.1% aprotinin, 10 mM β-mercaptoethanol, 160 unit/mL RNaseOUT) by gently flicking the tube.

8. Add 0.5 mL cold nuclei lysis buffer (10 mM HEPES, pH 7.6, 1mM DTT, 7.5 mM MgCl2, 0.2 mM EDTA, 0.3 M NaCl, 1 M Urea, 1% NP-40, 0.5 mM PMSF, Proteinase inhibitor cocktail, 0.1% pepstatin A, 0.1% aprotinin, 10 mM β-mercaptoethanol, 160 unit/mL RNaseOUT).

9. Gently vortex for 2 × 2 sec, incubate on ice for 2 min, centrifuge at 14,000 rpm at 4°C for 2 min to separate the chromatin fraction and the nucleoplasmic fraction.

10. Treat the supernatant (nucleoplasmic fraction) with proteinase K at 37°C for 1 hr, followed by traditional acidic phenol/chloroform extraction and ethanol precipitation for RNA.

11. Gently rinse the chromatin pellet twice with 0.5 mL cold wash buffer and then dissolve in 1 mL Trizol for RNA extraction.

12. Refer to *Section III-1* for RT-qPCR of scaffold RNAs.

## Competing interests

The authors declare that they have no competing interests.

## Authors’ contributions

HZ performed the experiments. KT participated in siRNA sequence analysis. BW participated in Chop PCR and siRNA RT-qPCR. C-GD participated in Chop PCR. ZL participated in Chop PCR. J-KZ conceived of the study and wrote the manuscript with HZ. All authors read and approved the final manuscript.

## Supplementary Material

Additional file 1: Figure S1 Examples of methylation-sensitive Chop PCR and Chop qPCR. DNA methylation at a group of transposon loci were examined by Chop PCR or Chop qPCR. Mutants were compared with the wild type Arabidopsis (*Col-0*). For BsmF I, BstBI, or Hae III digestion, a DNA sequence from *SKP1* without the restriction sites was examined as loading control. For HpyCH4 IV, 5TE27040 was amplified as non-digestion control. **[A]** Chop PCR. Restriction enzymes BsmF I, HpyCH4 IV, BstB I, and Hae III are indicted on the right. The examined loci are annotated on the left. **[B]** Chop qPCR. Hae III was used for digestion. Methylation levels, as indicated by qPCR signals, in the mutants were relative to those in *Col-0. SKP1* was used as loading control. Error bars indicate SD, n ≥ 3. Primers are listed in Table S3. **Figure S2.** Detection of Arabidopsis miRNAs by using TaqMan Small RNA Assay. Aerial portions of plants grown with (P deficient) or without (control) phosphate deficiency stress were examined. Each RT reaction used 80 ng total RNA and *snoR101* was used as internal control. Error bars indicate SD, n = 3. **Figure S3.** Quality control of nuclei fractionation. qPCR detection of genomic DNA (gDNA) in the chromatin-associated fraction (pellet) and the chromatin-free fraction (supernatant) after nuclei fractionation. *SKP1* gDNA levels in the chromatin-free fraction were presented relative to those in chromatin-associated fraction. Error bars indicate SD, n = 3. Primers are listed in Table S3. (PDF 363 kb) Click here for file

Additional file 2: Table S1 Methylation-sensitive restriction enzymes for Chop PCR. **Table S2.** Arabidopsis 24nt siRNAs detected by using TaqMan Small RNA Assays. **Table S3.** Primers used for PCR/qPCR in this report. Click here for file
