# Supplementary figures and images for: Protocol: a beginner’s guide to the analysis of RNA-directed DNA methylation in plants
Source: Plant Methods. 2014 Jun 14;10:18. doi: 10.1186/1746-4811-10-18 (PMC4065543; doi:10.1186/1746-4811-10-18)

Figure S1

[A]

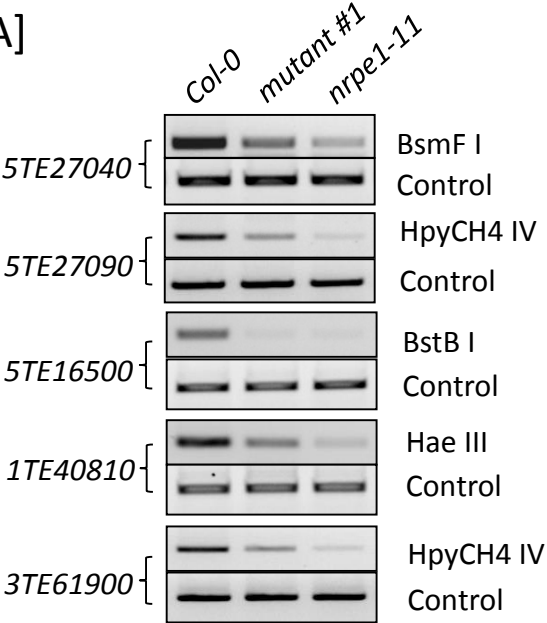

[B]

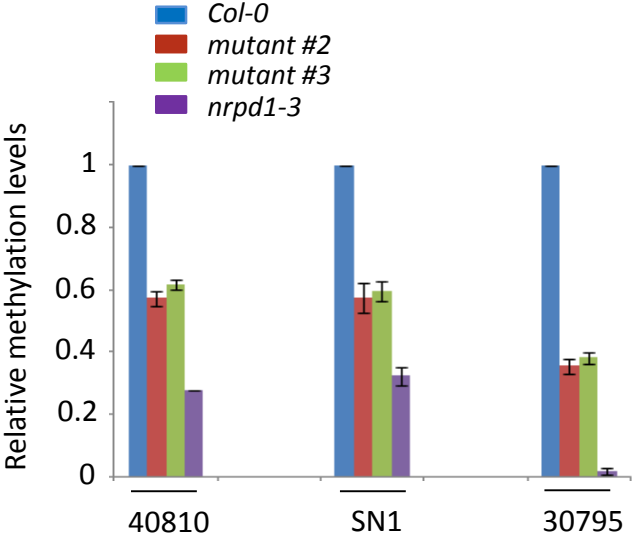

Figure S2

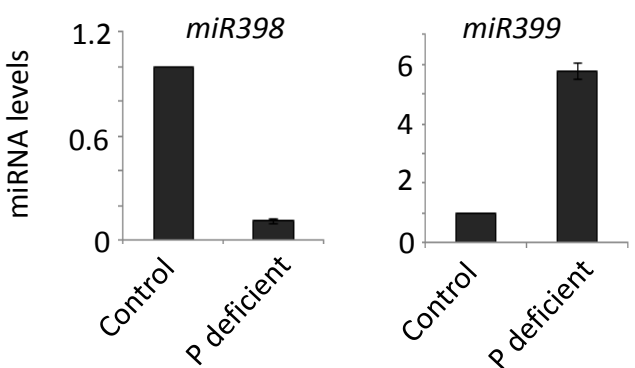

**Figure S3**

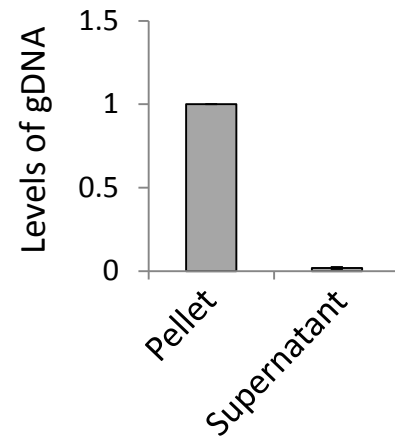

Supplement: Additional file 1: Figure S1 — Examples of methylation-sensitive Chop PCR and Chop qPCR. DNA methylation at a group of transposon loci were examined by Chop PCR or Chop qPCR. Mutants were compared with the wild type Arabidopsis (Col-0). For BsmF I, BstBI, or Hae III digestion, a DNA sequence from SKP1 without the restriction sites was examined as loading control. For HpyCH4 IV, 5TE27040 was amplified as non-digestion control. [A] Chop PCR. Restriction enzymes BsmF I, HpyCH4 IV, BstB I, and Hae III are indicted on the right. The examined loci are annotated on the left. [B] Chop qPCR. Hae III was used for digestion. Methylation levels, as indicated by qPCR signals, in the mutants were relative to those in Col-0. SKP1 was used as loading control. Error bars indicate SD, n ≥ 3. Primers are listed in Table S3. Figure S2. Detection of Arabidopsis miRNAs by using TaqMan Small RNA Assay. Aerial portions of plants grown with (P deficient) or without (control) phosphate deficiency stress were examined. Each RT reaction used 80 ng total RNA and snoR101 was used as internal control. Error bars indicate SD, n = 3. Figure S3. Quality control of nuclei fractionation. qPCR detection of genomic DNA (gDNA) in the chromatin-associated fraction (pellet) and the chromatin-free fraction (supernatant) after nuclei fractionation. SKP1 gDNA levels in the chromatin-free fraction were presented relative to those in chromatin-associated fraction. Error bars indicate SD, n = 3. Primers are listed in Table S3. (PDF 363 kb) [file 1746-4811-10-18-S1.pdf]
