# Supplementary material for: Protocol: a beginner’s guide to the analysis of RNA-directed DNA methylation in plants
Source: Plant Methods. 2014 Jun 14;10:18. doi: 10.1186/1746-4811-10-18 (PMC4065543; doi:10.1186/1746-4811-10-18)
Supplement: Additional file 2: Table S1 — Methylation-sensitive restriction enzymes for Chop PCR. Table S2. Arabidopsis 24nt siRNAs detected by using TaqMan Small RNA Assays. Table S3. Primers used for PCR/qPCR in this report. [file 1746-4811-10-18-S2.pdf]

Supplemental Information

Protocol: A beginner's guide to the analysis of RNA-directed DNA methylation in plants

Huiming Zhang[1,*], Kai Tang[1], Bangshing Wang[1], Cheng-Guo Duan[1], Zhaobo Lang[1], Jian-Kang Zhu [1,2,*]

[1]Department of Horticulture and Landscape Architecture, Purdue University, West Lafayette, IN 47907, USA

[2]Shanghai Center for Plant Stress Biology, Shanghai Institute of Biological Sciences, Chinese Academy of Sciences, China.

*To whom correspondence may be addressed. Email: Zhang, Huiming (zhang731@purdue.edu) or Zhu, Jian-Kang (jkzhu@purdue.edu).

Supplemental Experimental Procedures

Plant Materials

All *Arabidopsis thaliana* used in this study is of the Columbia ecotype (*Col-0*). Mutants including *nrpd1-3* (SALK_128428C) and *nrpe1-11* (SALK_029919C) are from ABRC. Plants were grown in ½-strength MS medium with 1.5% sucrose and 0.7% agar. Phosphate deficiency treatment was performed as described [1].

Supplemental Figure Legends:

Figure S1. Examples of methylation-sensitive Chop PCR and Chop qPCR. DNA methylation at a group of transposon loci were examined by Chop PCR or Chop qPCR. Mutants were compared with the wild type Arabidopsis (*Col-0*). For BsmF I, BstBI, or Hae III digestion, a DNA sequence from *SKP1* without the restriction sites was examined as loading control. For HpyCH4 IV, 5TE27040 was amplified as non-digestion control. [A] Chop PCR. Restriction enzymes BsmF I, HpyCH4 IV, BstB I, and Hae III are indicted on the right. The examined loci are annotated on the left. [B] Chop qPCR. Hae III was used for digestion. Methylation levels, as indicated by qPCR signals, in the mutants were relative to those in *Col-0*. *SKP1* was used as loading control. Error bars indicate SD, $n \geq 3$. Primers are listed in Table S3.

Figure S2. Detection of Arabidopsis miRNAs by using TaqMan Small RNA Assay. Aerial portions of plants grown with (P deficient) or without (control) phosphate deficiency stress were examined. Each RT reaction used 80 ng total RNA and *snoR101* was used as internal control. Error bars indicate SD, $n = 3$.

Figure S3. Quality control of nuclei fractionation. qPCR detection of genomic DNA (gDNA) in the chromatin-associated fraction (pellet) and the chromatin-free fraction (supernatant) after nuclei fractionation. *SKP1* gDNA levels in the chromatin-free fraction were presented relative to those in chromatin-associated fraction. Error bars indicate SD, $n = 3$. Primers are listed in Table S3.

Supplemental Tables:

Table S1. Methylation-sensitive restriction enzymes for Chop PCR

Enzyme	Restriction sites (5' to 3')	Arabidopsis RdDM marker loci (coordinates)
Hae III	CC*GG	*AtSN1* (Chr: 15794808 -15794619)<br>*1TE40810* (Chr1: 12530263 – 12530392)<br>*5TE30795* (Chr5: 8511092 – 8511269)
HpyCH4 IV	A*CGT	*5TE27090* (Chr5: 7490952 – 7491104)<br>*3TE61900* (Chr3: 15221816 – 15221989)
BstB I	TT*CGAA	*5TE16500* (Chr 5: 4561191 -4561364)
BsmF I	GGGAC$(N)_{10}$*	*5TE27040* (Chr5: 7478632 – 7478460)
Hpa II	C*CGG	*solo LTR* (Part of the Intergenic region between At5g27845 and At5g27850) [2]
Msp I	C*CGG	*solo LTR*
Alu I	AG*CT	*solo LTR*<br>*IGN5* (Intergenic region between At4TE10770 and At4TE10775) [3]
McrBc	G/A-^{m}C$(N)_{40-3000}$-G/A-^{m}C	*GP1* (At4g03650) [4]<br>*MU1* (At4g08680) [4]

Asterisks (*) denote restriction sites. McrBc is different from other listed enzymes in that it cleaves methylated- but not unmethylated DNA sequences.

Table S2. Arabidopsis 24nt siRNAs detected by using TaqMan Small RNA Assays

Name	Genomic loci (reference)	24nt siRNA sequences
AtSN1	At3TE63860 [3]	AUCUGAGAGAUUUACCACUGGGCC
siR1003	5S rDNA repeats [5,6]	AGACCGUGAGGCCAAACUUGGCAU
solo LTR	Part of the Intergenic region between At5g27845 and At5g27850 [2]	AGCUAAGUAAGCUCUACUAUAUAU
Cluster 2	Part of the intergenic region between At1g13195 and At1g13200 [7,8]	UAACUUGGAUACUGUGAAUGAUGC
IGN5B	At4TE10775 [3]	AUGUCGGCCAAUCUUCUUGAUUGU
5TE27040	At5TE27040 [9]	AUCAGCACAUCUACCUAGGAGAUU

Table S3. Primers used for PCR/qPCR in this report

**chop-PCR primers**	
5TE27040-MSD-F	TTAGAGGCGGGACCCATACCTAAT
5TE27040-MSD-R	GAGCTGTTGGCCCAATGGTAAA
5TE27090-MSD-F	AGTGGCCATATGTACTCGTGTG
5TE27090-MSD-R	AGCTCTACATCTCTCTGAGGCA
TE16500-MSD-F	TCAAGTGAAGGTGCTAGGTTCG

TE16500-MSD-R	ACATTTCCTCCGCTCCGGTTTA
1TE40810-MSD-F	ACACAAAGGCCCTACCTACA
1TE40810-MSD-R	ACGGCACAAGGGCTAGTATT
3TE61900-MSD-F	GCGTTTGTGTTCGAATCGAGGT
3TE61900-MSD-R	ACCAACGTGTTATTGCCCAGTG
solo LTR- F	ATAAAACTCGAAACAAGAGTTTTCTTATTGCTTTC[3]
solo LTR- R	TAATGGTATTATTTTGATCAGTGTTATAAACCGGA[3]
IGN5- F	TCCCGAGAAGAGTAGAACAAATGCTAAAA[3]
IGN5- R	CTGAGGTATTCCATAGCCCCTGATCC[3]
GP1- F	ACAGTGCCACAGTTGAGCAG[10]
GP1- R	CAGAAAAATACTCGGTGCCAAT[10]
MU1- F	GTGGATATACCAAAAACACAA[10]
MU1- R	CTTAGCCTTCTTTTCAATCTCA[10]
**Chop-qPCR primers**	
SN1-qF	ATCTGGAAGTTCAGGCCCAA
SN1-qR	TGCTGGATTCGAGACACGTT
5TE30795-qF	TGGAAGTACTCGGTTCGAGCAA
5TE30795-qR	AGCTGCGTTTGAGTGGAGAGTA
1TE40810-MSD-F	ACACAAAGGCCCTACCTACA
1TE40810-qR	TCTTTCAGGTGGAGGTGTTGGA
SKP1-qF	GATCCGCACAACGTTCAACA

SKP1-qR	GAAACTGGTTGCTTGCTGCT
**qPCR for scaffold RNAs**	
SN1C-GSP	CCTTTCCAAGACACCATCTCAACAAC
SN1C-qF1	CACTCCAGCTCCATGTCATT
SN1C-qR1	CGACTCCCATAAGTAACGAG
IGN5A-GSP	CTGAGGTATTCCATAGCCCCTGATCC
IGN5A-qF	GCGGCCCAATAACCAACAAA
IGN5A-qR	TTGGGCCGAATAACAGCAAG
IGN15-GSP	CGGAAAAGGTAAGGTGGTTGGAAAA
IGN15-qF	CGGACTTGACCAATCCAATAAA
IGN15-qR	AAAGGTAAGGTGGTTGGAAA
IGN17-GSP	AACCCTAGCCTTTCATTAAAACCCTCTC
IGN17-qF	CCCTCTCTGAATTCATCTCTAGTATTT
IGN17-qR	TGTAGCTGCTTTGTCTGATATGT
Act2-GSP	CTAAGCTCTCAAGATCAAAGGC
ACT2-qF	TGTGTGACAAACTCTCTGGG
ACT2-qR	GGCATCAATTCGATCACTCAG

Supplemental References:

1. Hsieh LC, Lin SI, Shih AC, Chen JW, Lin WY, Tseng CY, Li WH, Chiou TJ: **Uncovering small RNA-**

mediated responses to phosphate deficiency in Arabidopsis by deep sequencing. *Plant Physiol*. 2009, **151:** 2120–2132.

2. Huettel B, Kanno T, Daxinger L, Aufsatz W, Matzke AJ, Matzke M: **Endogenous targets of RNA-directed DNA methylation and Pol IV in Arabidopsis**. *EMBO J.* 2006, **25:** 2828–2836.

3. Wierzbicki AT, Haag JR, Pikaard CS: **Noncoding transcription by RNA polymerase Pol IVb/Pol V mediates transcriptional silencing of overlapping and adjacent genes.** *Cell* 2008, **135**:635–648.

4. Lippman Z, May B, Yordan C, Singer T, Martienssen R: **Distinct mechanisms determine transposon inheritance and methylation via small interfering RNA and histone modification.** *PLoS Biol.* 2003, **1:** e67.

5. Preuss SB, Costa-Nunes P, Tucker S, Pontes O, Lawrence RJ, Mosher R, Kasschau KD, Carrington JC, Baulcombe DC, Viegas W, Pikaard CS: **Multimegabase silencing in nucleolar dominance involves siRNA-directed DNA methylation and specific methylcytosine-binding proteins**. *Mol. Cell* 2008, **32:** 673–684.

6. Zilberman D, Cao X, Johansen LK, Xie Z, Carrington JC, Jacobsen SE: **Role of Arabidopsis ARGONAUTE4 in RNA-directed DNA methylation triggered by inverted repeats**. *Curr Biol*. 2004, **14**: 1214-1220.

7. Xie Z, Johansen LK**,** Gustafson AM**,** Kasschau KD**,** Lellis AD**,** Zilberman D**,** Jacobsen SE**,** Carrington JC**: Genetic and functional diversification of small RNA pathways in plants.** *PLoS Biol.* 2004, **2:** E104.

8. Pontier D, Yahubyan G, Vega D, Bulski A, Saez-Vasquez J, Hakimi MA, Lerbs-Mache S, Colot V, Lagrange T: **Reinforcement of silencing at transposons and highly repeated sequences requires the concerted action of two distinct RNA polymerases IV in Arabidopsis.** *Genes Dev*. 2005, **19**: 2030-2040.

9. Zhang H, Tang K, Qian W, Duan CG, Wang B, Zhang H, Wang P, Zhu X, Lang Z, Yang Y, Zhu JK: **An Rrp6-like Protein Positively Regulates Noncoding RNA Levels and DNA Methylation in Arabidopsis**. *Mol Cell* 2014, **54**: 418-430.

10. He XJ, Hsu YF, Pontes O, Zhu J, Lu J, Bressan RA, Pikaard C, Wang CS, Zhu JK. **NRPD4, a protein related to the RPB4 subunit of RNA polymerase II, is a component of RNA polymerases IV and V and is required for RNA-directed DNA methylation.** *Genes Dev*. 2009, 23: 318-330.
